# Post-Bariatric Surgery Complications and the Role of Endoscopic Intervention

**DOI:** 10.3390/diagnostics16142220

**Published:** 2026-07-16

**Authors:** Giuliana Mertz, Brandon Stahl, William Schu, Kumael Jafri, Jessica Zaman

**Affiliations:** Albany Medical Center, 43 New Scotland Avenue, Albany, NY 12208, USA

**Keywords:** endoscopy, obesity, bariatric surgery, endoscopic evaluation, therapeutic endoscopy, Roux-en-Y gastric bypass, sleeve gastrectomy, metabolic surgery, weight management

## Abstract

The prevalence of obesity across the world, especially in the United States, remains at epidemic proportions, creating significant health complications in these patients and posing challenges for overcoming numerous related conditions. Over the years, the surgical approach to the management and treatment of obesity and associated morbidities has continued to increase in use and has been an effective treatment method. Various approaches to surgical management include Roux-en-Y gastric bypass (RYGB), sleeve gastrectomy (SG), adjustable gastric banding, biliopancreatic diversion with duodenal switch (DS), and endoscopic intragastric balloons. Endoscopy has remained a mainstay approach to evaluating post-operative changes and complications following these procedures. In this scoping review, we discuss the indications and optimal timing for endoscopic evaluation post-bariatric surgical intervention, common endoscopic findings, endoscopic therapeutic techniques and challenges, as well as future directions in the field of post-operative endoscopy. We conclude that while some complications require immediate surgical or radiologic intervention prior to endoscopy, other complications may be evaluated with endoscopy.

## 1. Introduction

The prevalence of obesity across the world, and especially in the United States, remains at epidemic proportions. Significant health complications due to obesity-related comorbidities pose a challenge both in medical management and from a health economic standpoint. Over the years, surgical treatment of obesity and associated comorbidities has emerged as a consistent and effective tool in treating this disease. Bariatric surgery has been a mainstay approach of treatment for obesity. Popular surgical approaches include the Roux-en-Y gastric bypass (RYGB), sleeve gastrectomy (SG), adjustable gastric banding, biliopancreatic diversion with duodenal switch (DS), and endoscopic intragastric balloons. However, bariatric surgery does not come without risk, spanning over various post-operative time periods. Alongside other diagnostic tools, endoscopy is a standard approach for post-operative evaluation after bariatric surgery. In this scoping review, we discuss the indications and optimal timing for endoscopic evaluation post-bariatric surgical intervention, common endoscopic findings, endoscopic therapeutic techniques, and challenges, as well as future directions in the field of post-operative endoscopy through PubMed searches of recent articles and studies within the field of bariatric surgery, post-bariatric surgery endoscopy, and complications following bariatric surgery. The quality of evidence supporting endoscopic evaluation and management of post-bariatric surgery complications varies considerably across disease states. While some topics are supported by systematic reviews and larger observational studies, many diagnostic and therapeutic approaches are based primarily on retrospective cohort studies, case series, and expert opinion due to prospective comparative data being limited. This scoping review will investigate more deeply the more commonly used approaches to bariatric surgery, sleeve gastrectomy, and Roux-en-Y gastric bypass, with some mention of other bariatric surgery approaches.

## 2. Overview of Bariatric Procedures

From 2013 to 2023, RYGB accounted for approximately 26% (*n* = 550,833) of all bariatric procedures performed in the United States [1]. In RYGB, a small proximal gastric pouch is anastomosed to a Roux (alimentary) limb of the jejunum to bypass the distal stomach, duodenum, and proximal jejunum. Gastrojejunostomy and jejunojejunostomy are created, which separate biliopancreatic secretions from alimentary flow until they meet in the common limb [2].

During that same timeframe, SG accounted for about 68% (*n* = 1,441,481) of all bariatric procedures [1]. During an SG, a longitudinal resection of the greater curvature of the stomach converts the stomach into a narrow tubular conduit and is closed with staples. The pylorus is preserved during this procedure along with the rest of the gastrointestinal tract [3].

Adjustable gastric banding has become less common (~4%; *n* = 80,017) with the emergence of RYGB and SG [1]. This procedure involves the placement of an inflatable silicone band around the proximal stomach to create a small pouch that aids in restricting food intake. A small subcutaneous port is created, which allows the band to be loosened or tightened. The band stoma is visible during endoscopy as a narrowing below the gastroesophageal junction, but access to the stomach and duodenum remains unchanged [4].

Biliopancreatic diversion with duodenal switch (DS) has also seen a decline in frequency (~1.4%; *n* = 29,908) due to its technical complexity and severe long-term nutritional complications [1]. This procedure is a combination of an SG and division of the duodenum. This division creates an alimentary limb and a biliopancreatic limb that meet in a short common channel, causing food to bypass approximately 75% of the small intestine [5].

With the altered anatomy after these procedures, there are implications for the use and utility of endoscopic interventions for post-operative management. Adjuvant techniques such as balloon endoscopy, push endoscopy, or laparoscopic-assisted endoscopy may be necessary to properly evaluate excluded bowel and remnant anatomy. Careful considerations such as scope diameter and insufflation pressures need to be kept in mind when dealing with smaller anastomosis or higher-pressure systems, such as the tubular stomach formed after SG. Endoscopy remains an important tool for the surveillance and management of post-bariatric patient populations.

## 3. Indications for Endoscopy Post-Bariatric Surgery and Common Endoscopic Findings by Surgery Type

Indications for endoscopic evaluation of post-bariatric surgery complications vary based on surgical anatomy implications, timing, and description of clinical presentation.

Clinical presentations that necessitate endoscopic evaluation can be classified based on the expected presentation of symptoms. Early post-operative changes may be characterized by a timeline of 0–30 days post-operation, although they may extend past this generalized period. Three complications often seen during this period include anastomotic leak, hemorrhage, and obstruction. Intermediate post-operative changes occur primarily between 1 and 6 months. Late post-operative changes often occur from 6 months to many years following bariatric surgery procedures. Complications organized by post-operative time period can be seen in Table 1.

### 3.1. Early Complications

Endoscopy in the early post-operative period (0–30 days) is indicated when trying to evaluate and/or treat acute intraluminal complications such as staple line or anastomotic leak, intraluminal hemorrhage, and obstruction. When used in combination with imaging, early endoscopic evaluation can provide a minimally invasive method of assessment and treatment.

**Staple line or anastomotic leaks** are estimated to occur in 0.75% of patients after SG and 1.5% of patients after RYGB and carry a mortality rate of 1.5% [6,7]. Patients with a body mass index (BMI) greater than 50 and those undergoing revisional surgery are at greater risk for leaks [8]. Anastomotic or staple line leaks should be suspected in patients in the 3–14-day range post-op who present with tachycardia, unexplained fever, persistent abdominal or shoulder pain, or decreased oral intake. Persistent tachycardia above 120 post-operatively is strongly suggestive of a leak [9]. Initial imaging will often include computed tomography (CT) or upper gastrointestinal (GI) contrast study and may show extraluminal contrast or a localized fluid collection near the staple line or gastrojejunostomy. The role of endoscopic evaluation is to delineate the size, location, and morphology of the defect along with mucosal viability in clinically stable patients who do not require urgent surgical intervention. In patients with hemodynamic instability, diffuse peritonitis, or evidence of uncontrolled sepsis, surgical management should not be delayed for endoscopic evaluation. Staple line or anastomotic leaks following bariatric surgery present endoscopically with a visible defect or opening at the staple line or anastomosis, most commonly at the proximal stomach near the angle of His in SG or at the gastrojejunal anastomosis in RYGB. The leak orifice is typically accompanied by surrounding mucosal inflammation and edema and may demonstrate a fistulous tract connecting the gastric lumen to peri-gastric collections. Downstream stenosis or stricture at the incisura angularis (in SG) or at the anastomotic site contributes to high intraluminal pressures that may propagate the leak. Necrotic or ischemic tissue may be visible surrounding the leak due to compromised blood supply from short gastric artery takedown, particularly in the proximal fundal region where the gastric wall is thinner. Early leaks (within 14 days) demonstrate acute inflammation, while late leaks (>4–6 weeks) and chronic fistulae show more established fistulous tracts with organized collections.

**Hemorrhage** will typically occur within 72 h of the operative period and should be suspected in patients with hematemesis, melena, hematochezia, or signs of hypovolemia on exam. The incidence of post-operative hemorrhage is estimated to occur in 0.5 to 5.8% of patients after bariatric surgery [10]. Intraluminal hemorrhage is almost 3 times more likely to occur when compared to extraluminal hemorrhage, and early differentiation is crucial [11]. Intraluminal hemorrhage will often present with melena, hematemesis, hematochezia, or blood from a nasogastric tube. Extraluminal hemorrhage should be suspected in those with a falling hemoglobin without signs of an overt gastrointestinal bleed or those with worsening abdominal distention. As long as the patient is hemodynamically stable, initial imaging will generally consist of a CT Angiography (CTA) of the abdomen and pelvis with IV contrast. Specifically, a multiphasic CTA (arterial and portal venous phases) to help with both characterizing anatomy and localizing the source of the bleed. Importantly, CTA has limited sensitivity for slow intraluminal hemorrhage if there is no active extravasation. With that in mind, CTA should not delay resuscitation or returning to the OR in hemodynamically unstable patients. Once the patient is hemodynamically stable, endoscopic evaluation can be both diagnostic and therapeutic in identifying the site of hemorrhage and guiding further management [10,11].

**Obstruction** will typically present on the order of days to weeks in the early post-operative period as emesis, inability to tolerate oral intake, early satiety, abdominal distention, or absence of flatus. The obstruction can be transient and secondary to edema or a mechanical obstruction. Early obstruction (within 30 days post-operatively) is primarily due to technical problems with the Roux limb [12]. Due to defects in the bowel mesentery after RYGB, internal hernias are another common cause of obstruction that is important not to miss, with an incidence of 2.5% in patients after RYGB [8,12]. Initial imaging for these patients will often include a CT of the abdomen and pelvis with IV contrast to delineate anatomy and assess for bowel wall enhancement, identify ischemia, and evaluate mesenteric vasculature. Endoscopic evaluation is warranted in patients when the obstruction is thought to be due to an intraluminal or anastomotic pathology, such as a phytobezoar or large food bolus, but should not delay surgical intervention if there is concern for mechanical or vascular compromise [13].

### 3.2. Intermediate Complications

Complications in the intermediate period following weight loss surgery include dysphagia, marginal ulcers, strictures, and fistulas. Treatment may involve surgical, endoscopic, or radiological procedures.

**Marginal ulcers** (MUs) typically occur within the first year after RYGB but can develop at any point after bypass. The incidence is as high as 18% [14]. Although not fully understood, the formation of MU is thought to be multifactorial and both nonmodifiable and modifiable. With RYGB anatomy, a lack of antral stimulation by food bolus passage leads to unopposed acid production. Without antral buffering, the jejunum is exposed to a high-acid environment, likely contributing to erosion and ulcer formation. Gastric pouch size appears to be linked to MU formation, with larger pouches containing more parietal cells. Ayuso et al. demonstrated that for every 5 cm^3^ increase in pouch size, there was a 2.4 times increase in the odds of ulcer formation [15]. Rates of MU formation with different anastomotic techniques and suture choices have been studied, with some groups demonstrating observational studies and systematic reviews suggesting an increase in MU formation with circular stapled anastomosis, as opposed to hand-sewn or linear stapled [16,17]. This is proposed to result from excess foreign material at the anastomosis, causing chronic inflammation and predisposing tissues to ulceration. Other modifiable risk factors such as smoking, diabetes, steroid use, H. pylori infection, and alcohol consumption have also been shown to have an association with marginal ulcers. Endoscopy can assist with ulcer diagnosis while simultaneously allowing for interventions for these patients, such as bleeding control and tissue biopsy. Marginal ulcers following bariatric surgery appear endoscopically as mucosal defects at the gastrojejunal anastomosis, typically located on the jejunal side of the anastomosis. In 76.7% of cases, the ulcer is found on the Roux limb side, though 50% may be located directly at the anastomosis itself. Notably, 24–25% of patients with marginal ulcers are asymptomatic and diagnosed only on routine surveillance endoscopy, highlighting the importance of endoscopic evaluation in this population.

Treatment for MUs often follows a stepwise approach. With uncomplicated index ulcers, conservative management with acid-suppression medication, stomach lining coating agents such as sucralfate, and lifestyle modifications can be effective for ulcer regression. Evidence supporting this practice is limited and derives primarily from retrospective data, with one study suggesting that opening proton pump inhibitor capsules in RYGB patients improves absorption of the medication and enhances healing [18]. The majority of MUs heal with medical therapy; several retrospective studies found healing rates anywhere from 67 to 100% [19,20]. Interpretation of these rates is limited by differences in treatment protocols and follow-up practices among available studies. Endoscopic interventions have been described for chronic or refractory ulcers. Covering ulcers that fail medical therapy, including endoscopic suture imbrication or placing and placement of fully covered stents. However, available evidence is limited primarily to retrospective studies and case series, and optimal patient selection remains incompletely defined [21]. Reported endoscopic stent approaches are both viable options; approaches include endoscopic suture imbrication and placement of fully covered stents, with the goal of reducing acid exposure and promoting ulcer healing. In a retrospective study completed by Baroud et al., a population of 46 patients underwent endoscopic MU imbrication with either a tack device or overstitch suturing. Symptom resolution was reported in 70.6 and 75% of their patients had resolutions, respectively, although interpretation is limited by the retrospective design and lack of symptoms following endoscopic intervention in a comparison group [21]. Surgical intervention remains another mainstay for refractory ulcers, and options include revision of the gastrojejunal anastomosis, reversal of RYGB anatomy, or oversewing the ulcer edge.

**Dysphagia** is common after weight loss surgery and is seen after both RYGB and SG procedures. In a 2025 study by Saleh et al., rates of dysphagia after RYGB and SG were found to be 14.6% and 8.7%, respectively [22]. The etiology of dysphagia in these patients is broad and multifactorial. The restrictive aspects of these surgeries inherently lead to dysphagia in the intermediate post-op period. Failure to adhere to dietary recommendations and overeating can lead to poor pouch emptying and intolerance to food, a reason why nutritional education is critical in the pre-operative period. Similarly, delayed gastric emptying secondary to strictures, ulcers, small gastrojejunal (GJ) anastomosis, or early post-operative swelling can lead to dysphagia from partial obstruction. In some patients, dysphagia develops as a symptom of adjacent pathology, such as reflux, which is commonly encountered after weight loss surgery. Similarly, esophageal motility, either occult prior to surgery or de novo from altered drainage anatomy or damage to branches of the vagal nerve, can be a source of dysphagia in the bariatric surgery population. In a 2020 study by Miller et al., dysmotility was found in 13.7% of patients who had undergone RYGB surgery. Furthermore, it was demonstrated that increasing time since surgery was associated with increased risk of motility issues [23]. It is important to consider each of these possible sources during the evaluation of dysphagia.

Management of dysphagia is dependent on the primary cause, but upper endoscopy is a critical part of the workup. Behavioral modification and post-bariatric nutritional coaching are essential elements of treatment. Depending on the cause of dysphagia, endoscopy allows for interventions, such as dilations and oversewing.

**Anastomotic strictures** can develop any time after surgery, and the estimated incidence ranges from 0.4 to 6% [24]. Structure incidence is likely underestimated as a portion of patients are asymptomatic [25]. Strictures present with dysphagia, nausea, fullness, intolerance to certain foods, and emesis of undigested foods. Strictures present in the immediate post-operative period are often secondary to surgical technique and may require a return to the OR for revision; however, these are rare. The pathology of stricture formation is multifactorial. As in the case with all surgical anastomoses, tension, poor blood supply, and quality of tissue are critical in healthy anastomotic healing. Strictures can develop in the setting of GJ anastomosis inflammation, such as ulcers or chronic ischemia. Surgical techniques may have some influence on stricture development, but data are inconclusive. Some studies suggest circular staples have an increased risk of stricture formation, particularly with 21 mm size staplers [24,26].

Endoscopy is the mainstay of treatment for strictures. On endoscopic evaluation, strictures typically appear as narrowed gastrojejunal anastomoses < 10 mm in diameter, often accompanied by associated findings such as marginal ulceration, unraveled suture material, or edema. While medical therapy may be useful in the treatment of strictures secondary to ulcers, strictures often require dilation for symptom control. Dilation is often completed sequentially, starting with 8 mm balloons with a typical upper limit of 15 mm diameter balloons. Most strictures are successfully treated with a single intervention, but multiple dilations may be needed for complete resolution [27]. Success rates of endoscopic dilation range from 93% to 97% in reported series [28,29]. These data are derived primarily from observational studies and case series, which may limit their generalizability across different patient populations and treatment approaches.

**Fistulas** represent a rare but feared complication that can occur in the intermediate period after undergoing RYGB or SG. Incidence of fistula formation is poorly reported, but available studies report rates less than 5%, a similar incidence to leaks [30]. In both RYGB and SG, fistulas are theorized to be a sequela of contained leaks, but the pathophysiology of leak formation between these two procedures varies slightly. A standard SG results in a long linear staple line; fistulas usually result from leaks at this staple line. Studies have demonstrated that the tubular stomach created in SG has, on average, significantly increased basal pressures, likely as a result of an intact pylorus and a reduced ability for distension with higher pressures. Perforations and fistulas tend to occur near the gastroesophageal junction (GEJ), where this pressure is maximal [31,32]. The RYGB procedure involves multiple anastomoses, all of which can result in leaks and subsequent fistula formation. Gastrogastric (GG) fistulas are unique to RYGB given the proximity of the gastric pouch and the remnant stomach. As is the case with all staple lines and anastomoses, tension, poor blood supply, operative technique, and ulcers, as well as modifiable risk factors such as smoking, diabetes, and poor nutrition, can all contribute to leak and fistula formation. Patients with fistulas present with a variety of symptoms, including pain, abdominal bloating, failure of weight loss, pain with eating, fevers, nausea, diarrhea, and cutaneous fistulization [33,34].

Fistulas are often diagnosed with an upper GI series when contrast is seen extravasating from the lumen or, as in the case with GG fistulas, entering the remnant stomach. Endoscopy allows for categorizing these fistulas, and in patients where symptoms are persistent or when conservative measures do not result in closure, covered stents are commonly used to assist in closure. A meta-analysis by Okazaki et al. reported success rates in endoscopic stent placement for fistula and leak closure as high as 73% and 76.1% in SG and RYGB, respectively. These findings should be interpreted in the context of substantial heterogeneity among included studies and the predominance of non-randomized data. Additional means of fistula closure are discussed in subsequent sections [35].

### 3.3. Late Complications

Endoscopic evaluation for late complications of bariatric surgery is indicated when patients present with nausea and vomiting, gastroesophageal reflux, chronic diarrhea, dumping syndrome, nutritional deficiencies, generalized abdominal pain, and weight regain.

**Nutrient deficiencies** seen in post-bariatric surgery patients are often surgery-type dependent. As noted by Gasmi et al., nutrient deficiencies, including iron, selenium, and vitamin B12, are often seen more commonly following adjustable gastric banding and SG, whereas nutrient deficiencies of essential vitamins, minerals, and trace elements are more commonly seen in patients who underwent RYGB and duodenal switch [36]. According to Goel et al., in a multicentric study of 11,568 patients, nutritional deficiencies were seen in 0.75% of study participants and included anemia, hypoproteinemia, and macronutrient deficiencies. The timeline of diagnosis of these nutrient deficiencies ranged from the first three months to after one year post-surgery and was associated with a higher mortality [37].

Endoscopy for nutrient deficiencies is only routinely performed for those who are deficient in iron with hemoglobin changes concerning gastrointestinal bleeding. Otherwise, those who are experiencing micronutrient deficiencies are often monitored and supplemented with appropriate nutrient supplementation if they are experiencing these in isolation and not in conjunction with diarrhea concerning small bowel disease [38].

**Gastroesophageal reflux disease (GERD)** is defined as pH-metry results correlating with patients’ symptomatology, including heartburn, regurgitation, and atypical nighttime symptoms of reflux or esophagitis on upper endoscopy. Up to one-third of post-bariatric surgery patients may experience new or worsening gastroesophageal reflux. De novo or worsening pre-existing GERD varies according to surgery type and is highest following SG and lowest in RYGB patients [39]. A recent multi-center study performed by Dowgiałło-Gornowicz et al. in 2025 examined the incidence of de novo GERD ten years post-bariatric surgery and found that up to one-quarter of patients have GERD at the 10-year period following bariatric surgery, highlighting the importance of mindfulness of providers caring for patients after bariatric surgery [40]. The SLEEVEPASS trial comparing 10-year post-operative outcomes after SG and RYGB found 31% of SG patients had esophagitis as opposed to 7% of gastric bypass patients [41]. There are also concerns of elevated risk of Barrett’s esophagus in following SG patients compared to others, although available evidence is largely observational, and the magnitude of this risk remains incompletely defined [42].

GERD presenting after bariatric surgery is most often managed with lifestyle modification, including diet modification focusing on portion control, avoidance of triggers, and continued weight loss, and proton pump inhibitors. However, gastroesophageal reflux that is worsening or presenting with alarm symptoms, including dysphagia, odynophagia, unexplained weight loss or iron deficiency anemia, gastrointestinal bleeding, or persistent vomiting, must be evaluated endoscopically. Endoscopic variables to assess include previously discussed changes to anastomoses or pouch size, distal limb obstruction, or other anatomic variables [38]. We can conclude that endoscopy evaluating for GERD in post-bariatric surgery patients is indicated in instances of alarm symptoms or for worsening or unrelenting GERD to evaluate conditions including sleeve stenosis, hiatal hernia, or pouch dilation.

**Weight regain** is a common complication of bariatric surgery, with some reports noting up to 1 in 6 post-surgical patients experiencing 10% or greater weight regain from nadir [43]. Time from operation, amount of weight regained, and associated symptoms are highly varied [44]. The degree of weight regain is often surgery-type-dependent and is higher in SG patients as opposed to RYGB [45].

Management of weight regain begins with lifestyle modification and careful nutritional and behavioral review. Factors associated with weight regain include carbohydrate-heavy, food-rich dietary patterns, emotional eating, increased portion size, food urges, binge eating, loss of control/disinhibition when eating, and genetics that are positively associated with weight regain. Evaluating for genetic factors, gastrojejunal stoma diameter, and gastric volume following SG may improve weight regain after surgery [43]. Endoscopic evaluation is not the first line in the evaluation and management of those who experience weight regain during the post-operative period. However, complications of bariatric surgery, including inaccurate SG or pouch sizing, poorly calibrated anastomoses, and complications with gastric banding, may contribute significantly to weight regain and require endoscopic evaluation with surgical or advanced endoscopic revision or repair [44].

**Abdominal pain**, whether with an explainable cause or idiopathic in nature, has been reported in up to 20–30% of post-bariatric surgery patients [46,47]. Symptoms vary by surgery type, with RYGB patients demonstrating a higher risk of chronic abdominal pain after surgery [47]. Common causes of abdominal pain have been previously explored in this review, including internal hernias, marginal ulcers, biliary disease, anatomic strictures, or intussusception. Some patients, particularly those who have undergone RYGB, experience unexplained chronic abdominal pain [48].

Post-operative abdominal pain is the most common endoscopically evaluated symptom following bariatric surgery, as the etiologies of abdominal pain are numerous and may be serious in nature. However, endoscopy may only provide an explanation for abdominal pain in up to 34% of cases [49]. It is important to supplement endoscopy with clinical history and other forms of imaging modalities, including abdominal CT, ultrasound, upper GI series, or imaging of the biliopancreatic tract. Patients with signs of bowel obstruction, internal hernia, perforation, ischemia, or hemodynamic instability require urgent surgical evaluation, and endoscopy should not delay definitive management.

**Chronic diarrhea** is defined as four weeks or more of increased frequency of defecation or increased stool weight of loose/watery stools, with patients noting varied definitions of diarrhea itself, including looseness of stool, increased frequency, defecation urgency, or incontinence as definitions [50]. Chronic diarrhea can range greatly from 8% to 46% and varies depending on the type of surgery performed [51]. Etiologies of chronic diarrhea also vary and can include exocrine pancreatic insufficiency, small intestinal bacterial overgrowth (SIBO), and malabsorption.

Loose stools are not uncommon in biliopancreatic diversion and RYGB [52]. It can be persistent for many years after surgery, with up to 3% of patients having experienced chronic diarrhea five years post-operatively [53]. A diagnostic approach for chronic diarrhea must start with an investigation into other major etiologies, including infectious diseases, SIBO, and exocrine pancreas insufficiency, which may be performed through breath and stool testing. Endoscopy is appropriate for the investigation of chronic diarrhea when there is concern for etiologies, including SIBO or celiac disease, which require a small intestine biopsy [54].

**Dumping syndrome** is characterized by the rapid transition of chyme from the stomach to the small intestine and can result from the impact of bariatric surgery on gastrointestinal motility. Dumping syndrome can be further divided into either early dumping syndrome (10–30 min after a meal), characterized by abdominal cramping, nausea and vomiting, tachycardia, and diarrhea, or late dumping syndrome (1–3 h after a high-carbohydrate meal), characterized by episodes of hypoglycemia [55]. In SG, dumping can result from high pressure on the pylorus, which can increase the speed of chyme transport from the stomach to the duodenum. In RYGB, bypassing the pylorus and increasing transit speed into the small intestine can result in a high percentage of patients, up to 18.25%, experiencing post-operative dumping. This compares to lower rates of dumping in those who have undergone SG (about 5%) [56].

Similarly to other long-term complications, dumping syndrome must be evaluated for other etiologies of abdominal cramping, diarrhea, nausea, and vomiting. Investigation may include an oral glucose challenge test. Endoscopy may be used to investigate dumping syndrome, usually in cases where there is concern for other intestinal conditions, including SIBO or celiac disease, where biopsies are required for diagnosis. Management of dumping syndrome begins with lifestyle and behavioral modification. Patients are encouraged to have small meals frequently (4–6 small meals daily) and to wait at least 30 min before and after eating to drink liquids. Some medications may be used, including octreotide or acarbose. Lastly, surgical intervention may also be used to combat dumping syndrome, including revisions and reconstructions, which will be discussed in this review [57].

**Table 1:** Common complications following bariatric surgery by post-operative time period.

**Early Complications**	**Incidence**	**Typical Presentation**
Staple line or anastomotic leak	0.75% of patients following SG 1.5% of patients following RYGB [6,7]	Abdominal pain, tachycardia, fever, nausea, vomiting
Hemorrhage	0.5 to 5.8% of patients [10]	Tachycardia, hematemesis or melena, abdominal pain
Obstruction	2.5% of patients following RYGB [8,12]	Abdominal pain, nausea, vomiting, intolerance to PO intake
**Intermediate Complications**		
Marginal ulcers	Up to 18% of patients [14]	Gnawing, midepigastric pain worsening with eating, nausea, vomiting
Dysphagia	14.6% of patients following RYGB 8.7% of patients following SG [22]	Intolerance to PO intake, nausea, vomiting, regurgitation
Anastomotic strictures	0.4–6% of patients [24]	Nausea, vomiting, progressive dysphagia, abdominal pain, bloating, epigastric pain
Fistulas	<5% of patients [30]	Weight regain, abdominal pain, GERD, nausea, vomiting, GI bleed
**Late Complications**		
Nutrient deficiencies	0.75% of patients [37]	Varies by nutrient deficiency
Gastroesophageal reflux disease	Up to 31% of patients following SG 7% of patients following RYGB [41]	Heartburn, regurgitation, epigastric pain
Weight regain	Up to 17% of patients, depending on surgery type [43]	Increased weight by 10% or greater from the nadir
Chronic diarrhea	8–46% of patients, depending on surgery type [51]	Increased frequency of defecation or increased stool weight of loose/watery stool
Abdominal pain	20–30% of patients [46,47]	Dependent on the cause
Dumping syndrome	5–18.25% of patients, depending on surgery type [56]	abdominal cramping, nausea and vomiting, tachycardia, and diarrhea in early dumping syndrome; episodes of hypoglycemia in late dumping syndrome

### 3.4. Endoscopic Surveillance

Routine endoscopic surveillance is not an official recommendation for asymptomatic patients and has been controversial. Surveillance endoscopy is more appropriate for cases of patients who are at higher risk for pathologies, including Barrett’s esophagus after bariatric surgery, including SG, gastric remnant pathology in post-RYGB patients who are at high risk for gastric cancer, and for those with previously detected lesions.

**Barrett’s esophagus (BE)** can present de novo after SG, with literature demonstrating a 5.5% detection rate in SG patients 5–10 years after surgery and, in many cases, in patients without symptoms of GERD [58,59]. Many suggest routine screening in patients with prolonged reflux symptoms [58]. Recent American Society of Gastrointestinal Endoscopy (ASGE) guidelines also suggest screening in patients without GERD symptoms three years after SG and every five years subsequently [60].

**Gastric remnant pathology** is not routinely evaluated endoscopically unless there are significant risk factors for gastric malignancy or symptomatology that may be a result of gastric remnant bleeding or malignancy. However, endoscopic evaluation is difficult, and other imaging modalities may be attempted before endoscopy. Therefore, evaluation of patients for risk of gastric malignancies must be considered before bariatric surgery, especially RYGB [61].

## 4. Endoscopic Techniques and Tools

Over the last several decades, advancements in technology, endoscope design, and minimally invasive approaches to managing complications traditionally managed by surgery have prompted a surge in novel endoscopic techniques and interventions. Uses for these interventions are summarized in Table 2. These endoscopic modalities differ substantially in their indications, technical complexity, durability of response, and reported complication profiles. Selection of therapy is influenced by the type of defect, chronicity, anatomical location, and available expertise. Reported outcomes also vary widely across studies, reflecting heterogeneity in patient populations and procedural techniques. Direct comparisons between endoscopic modalities remain limited, as most available studies evaluate individual techniques in selected patient populations rather than comparing interventions head-to-head. Consequently, treatment selection is frequently guided by defect characteristics, timing of presentation, local expertise, and institutional experience, rather than high-quality comparative evidence.

**Stenting and balloon dilation** are durable interventions for anastomotic stricture, leak, and fistula control. Endoscopic balloon dilation is the standard of care for the management of strictures. Endoscope selection is based on anticipated interventions. Dilations are carried out via fixed balloons or over wire balloons depending on user preference, and concurrent fluoroscopic monitoring may be used as well. Balloon choice is based on structure size but typically ranges from 5 mm to 18 mm. Dilation is held for 60 s with a 60 s rest time between each dilation. At the end of the procedure, patency and adequate dilation are confirmed with endoscopic traversal. Patients may require repeat dilations, but most have improved symptoms after a single procedure. While other means of dilation exist, such as Savary-Gilliard bougie dilation, balloon dilation remains the most common among interventionalists.

Stent choice depends on the pathology being addressed. For fistulas or leaks, covered stents are best as they impede the passage of enteric contents, whereas permeable stents are preferred for strictures. Due to the diameter of pre-deployed self-expanding stents, placement requires larger working channels than typical diagnostic scopes. Therapeutic gastroscopes have larger channels that range from 3.7 to 6 mm in diameter. Prior to stent placement, endoscopy confirms location and size. Through the scope, wires can be used to traverse the lesion if dealing with a tight stricture. Stents are deployed either over the wire or directly from the endoscope. As with balloon dilation, fluoroscopy is often used in conjunction. When using fully covered stents, providers recommend suturing the stents in place to reduce the risk of migration. This is particularly important in stents placed for leaks or fistulas. With strictures, stents are less likely to migrate as the external force of the stricture keeps the stent in position.

In addition to more traditional stents, in some cases, transmural double-pigtail stents can be used to internally drain an abscess or a leak [62]. In these cases, a 7- or 10-French double-pigtail biliary stent is placed endoscopically through the site of perforation. This approach has most commonly been described in the setting of post-bariatric surgery leaks, with reported technical success dependent on early intervention and adequate cavity drainage, as demonstrated in a sleeve gastrectomy leak series [62]. Depending on the timing of intervention (early or late with regard to suspected perforation) and degree of containment of the perforation, internal drainage can be done independently or in conjunction with surgical washout or percutaneous drainage. Outcomes appear to be more favorable in contained leaks identified early in the clinical course, where delayed intervention and larger collections are associated with prolonged drainage duration and increased likelihood of adjunctive procedures. Consideration must also be given to nutrition, as internal drainage is not compatible with oral feeding. Total parenteral nutrition (TPN) and feeding via gastrojejunostomy/jejunostomy tube are the most common modes of nutritional supplementation in these patients.

**Endoscopic suturing** is technically difficult but allows for revision of bariatric anastomoses and, in some circumstances, can be used for fistula or perforation closure. Across published series, transoral outlet reduction (TORe) has demonstrated improvement in dumping symptoms and modest weight loss, although outcomes vary depending on technique, anatomy, and endoscopist experience [63,64,65]. As previously discussed, a dilated or excessively widened GJ anastomosis can lead to weight gain and dumping after RYGB due to loss of restriction and increased speed of transit into the small intestine. Transoral outlet reduction demonstrates consistent improvement in dumping symptoms while the impact on weight loss is more modest and variable [63,64]. Systematic reviews suggest that symptomatic improvement is more consistent than sustained weight reduction, with significant variability in long-term outcomes [63,65]. A thorough history and physical in conjunction with screening with upper endoscopy and quality of life surveys for dumping are necessary for accurate work-up [65]. The most common modality is the OverStitch device by Boston Scientific, which allows for full-thickness suturing via a therapeutic endoscope. The device has an integrated suture and allows for automated throwing and securing of sutures. Many recommend combining endoscopic suturing with argon plasma coagulation for more durable outcomes [66]. Endoscopic hand suturing (EHS) is an alternative approach but requires manual manipulation of a standard suture needle via endoscopic instruments. EHS is less commonly used for gastric suturing as it requires an overtube for suture advancement. EHS is more commonly utilized for esophageal suturing after esophageal mucosal resections or peroral endoscopic myotomy (POEM) procedures.

**Over-the-scope** clipping utilizes either traumatic or atraumatic jaw-like clips, which are placed on the endoscope extracorporeally and advanced with the scope until deployment. When fistulas fail to close via conservative treatment or interventions such as stent placement, over-the-scope clipping, or endoscopic vacuum therapy can be alternative options. Often paired with a grasping instrument in a working channel, tissue can be retracted into the jaws, and the clip can be deployed. These clips are a viable option in fistula closure, perforations, or hemostasis if smaller clips are insufficient. Reported success rates from a systematic review by Bartell et al. vary by defect characteristics, with closure rates of 55.8% for perforations and 72.6% for fistulas, and reduced efficacy in larger or chronic lesions when compared to acute defects [67]. Efficacy is highest in small, well-defined, non-epithelialized defects, with lower success in chronic or fibrotic lesions [67].

**Endoscopic vacuum therapy (EVT)** assists in the closure of intra-abdominal and intrathoracic cavities formed by perforations, leaks, or fistulae. Systematic reviews demonstrate high closure rates, particularly for esophageal leaks, though outcomes vary based on defect size, timing of initiation, and anatomical location [68]. EVT was developed in 2003 by Dr. Weidenhagen to treat rectal anastomotic leaks. The technique has since been applied to other pathologies, notably in the esophagus and stomach. First, a sponge is placed around a nasogastric tube (NGT) and secured in place. Sponges from negative pressure wound kits can be customized to the cavity being addressed and sutured to suction tubing. The sponge is then endoscopically placed in the defect or cavity. The proximal end of the NGT or suction tubing remains extracorporeal and is placed to suction. Portable vacuums designed for negative pressure wound control are typically used in outpatient settings. Sponges are routinely changed via endoscopy every 3–5 days until the cavity collapses and is obliterated [68]. Compared with stenting and clipping techniques, EVT is often associated with higher reported success in complex leaks but requires more frequent endoscopic interventions [68]. This process can take 2 weeks to several months, depending on the size of the cavity. While outcomes are generally positive, one drawback is prolonged hospitalization due to vac changes performed under anesthesia. This increased procedural burden and prolonged hospitalization represent key limitations compared with alternative endoscopic approaches such as stenting or over-the-scope clipping. When compared across endoscopic modalities, over-the-scope clipping is generally favored for small acute defects, while EVT and stent-based approaches are more commonly used in larger or complex leaks [67,68].

**Endoscopic sleeve gastroplasty** is an emerging, minimally invasive, and incisionless weight loss procedure. Meta-analyses report approximately 15–20% total body weight loss, although durability and long-term comparative efficacy versus surgical revision remain uncertain [69]. The mechanism of weight loss mirrors the restrictive mechanism of LSG. Either type of endoscopic suturing technique mentioned previously can be used: the Apollo Overstitch or Endoscopic Hand Suturing (EHS). The technique involves imbricating the greater curvature of the stomach via suture to achieve a 70% reduction in stomach volume. Outcomes are influenced by baseline BMI and suture pattern, and they appear to be durable procedural expertise, with heterogeneity across published studies [64,69].

**Across endoscopic modalities**, treatment selection is guided by defect type, chronicity, and anatomical location. Less complex or acute defects are more commonly managed with over-the-scope clips or stenting, whereas EVT is increasingly utilized for larger, more complex, or contaminated leaks requiring ongoing drainage [67,68]. Endoscopic suturing techniques are primarily applied in bariatric revision procedures, with variable efficacy depending on patient and procedural factors. Although each modality has demonstrated favorable outcomes in appropriately selected patients, current evidence does not clearly establish the superiority of one endoscopic approach over another for most post-bariatric complications, emphasizing the need for individualized treatment decisions and additional comparative studies.

## 5. Challenges and Considerations

When considering performing endoscopy in the post-bariatric surgery patient, there are several challenges and considerations. For example, altered anatomy of the gastrointestinal tract requires an appropriate selection of endoscopes and techniques. In addition, the training and expertise of the endoscopist is a consideration for the approach. Other risks include the post-operative risk of using an endoscope in a recently altered area. Lastly, it is important to consider using a multidisciplinary team to approach complications from bariatric surgery, as well as cost-effectiveness, high-risk groups, and post-bariatric surgery follow-up.

The **altered anatomy** following bariatric surgery calls for appropriate endoscopes and techniques to be used, especially in those with severely altered anatomy. In addition, the need for evaluation of the excluded segment (deep limb, excluded stomach, etc.) alters the approach to an otherwise routine esophagogastroduodenoscopy. If the intention is for evaluation of the gastric pouch and proximal anastomosis, a standard upper endoscope is appropriate. However, if the excluded segments must be evaluated, balloon-assisted enteroscopy is recommended [38]. Carbon dioxide insufflation is recommended over air insufflation to minimize the dilation of the deep limb and remnant stomach in RYGB, particularly in the 30-day post-operative window [70]. In patients with RYGB and biliary pathology, balloon-assisted enteroscopy may not be sufficient. In such cases, the remnant stomach can be accessed surgically (most frequently laparoscopically) to allow for endoscopic ultrasound (EUS) and endoscopic retrograde cholangiopancreatography (ERCP) [71]. In patients with chronic pathology who are unable to undergo surgical access to the remnant stomach, EUS-directed trans-gastric ERCP (EDGE) may be an option, in which case a gastro-gastric fistula is created between the gastric pouch and remnant stomach, allowing for ERCP through this endoscopically created fistula [72].

Although endoscopy is a generally safe procedure in the post-operative period, there are risks associated with endoscopy, and thus, precautions must be taken when performing endoscopy. As previously discussed, carbon dioxide insufflation is preferred over air insufflation to minimize time for reabsorption in the excluded portions of the gastrointestinal tract [70]. Specific precautions should be taken when entering the small bowel with the endoscope so as not to put excessive pressure on the staple lines [73]. Lastly, endoscopy poses a risk in the patient with significantly altered anatomy, and physicians should practice clinical judgment in these patients, especially in patients whose anatomy is not feasible to navigate, using more advanced approaches and those with more expertise.

**Training and expertise** are required for evaluation and therapeutic endoscopy on post-bariatric surgery patients. The American Gastroenterological Association (AGA) recommends that endoscopists should have “comprehensive knowledge of the indications, contraindications, risks, benefits, and outcomes of each of the endoscopic treatment techniques. They should also have knowledge of the risks and benefits of alternative methods such as surgical and interventional radiological-based approaches.” Also, AGA recommends that “Clinicians incorporating endoscopic management of bariatric/metabolic surgical complications into their clinical practice should have expertise in interventional endoscopy techniques, including but not limited to using concomitant fluoroscopy, stent deployment and retrieval, managing stenosis, and managing percutaneous drains” [70].

There are specific risks that occur when performing endoscopy on patients in the post-operative period following bariatric surgery. Risks include perforations, injury to the anastomoses, bleeding, and technical challenges. Perforation and anastomotic injury are most prevalent in the immediate post-operative period, and risks of perforation and injury may be mitigated with previously discussed carbon dioxide insufflation. Bleeding is another risk that may occur during endoscopy, more so related to thermal techniques, especially when close to anastomosis, leading to possible anastomotic necrosis or ischemia [74].

Multidisciplinary teams are of great importance when approaching complications after bariatric surgery. Per AGA guidelines, it is recommended that “clinicians performing endoscopic approaches to treat early major post-operative complications should do so in a multidisciplinary manner with interventional radiology and bariatric/metabolic surgery co-managing the patient. Daily communication is advised” [70]. Studies performed that examine the use of a multidisciplinary team, like that of Bullen et al. in 2019, have shown that using a multidisciplinary team resulted in a change in the plan for a significant number of patients, which this team interpreted as an improvement in the quality of care received by the patient [75]. As all patients should be treated for both physical and psychological side effects of bariatric surgery, multidisciplinary teams are often made up of bariatric surgeons, endoscopists, interventional radiologists, a registered dietitian, a primary care physician, and those who can address psychological concerns.

Regarding **cost,** currently, there is a paucity of literature and data reflecting direct cost analyses between surgical and endoscopic revisions for post-bariatric surgery complications. Most data presented currently are small, retrospective studies usually at single centers. However, although there is no explicit data comparison between the two approaches, it has been noted in limited studies that the endoscopic approach has been associated with shorter hospital stays and fewer adverse events [76,77]. Although endoscopic approaches, including stenting and over-the-stent clipping, for example, may be of high-cost burden, some data have shown that hospitalization costs may be lower. However, further data must be collected, and more detailed cost analyses must be performed to understand this more thoroughly.

When considering endoscopic intervention following bariatric surgery for complications, the risk associated must be taken into consideration, especially for **high-risk groups,** including those who have undergone abdominal or bariatric surgery prior to their most recent surgery, adults over the age of 65 years old, and those with multiple comorbidities. In addition, a large retrospective study found that older age and cardiovascular disease are associated with higher rates of morbidity and mortality following endoscopic weight loss management strategies, including sleeve gastrostomy [78].

There are many barriers to maintaining adequate **long-term follow-up**, resulting in less effective monitoring of patients for post-surgical complications. Long-term follow-up can be complicated, for example, by socioeconomic and access barriers, geographic distance, or psychosocial or behavioral factors. In the post-bariatric surgery population specifically, it has been noted, albeit often in single-center retrospective studies that are affected by poor generalizability, that long-term follow-up decreases overtime significantly from a noted 61.9% follow-up rate at 3 months to 6.5% at 24 months [79]. An improvement to this may include increasing the use of telehealth visits and increasing patient education on the risks of poor follow-up. The ASMBS recommends early follow-up in the immediate post-operative period with annual visits following [80].

## 6. Emerging Trends and Future Directions

### 6.1. Artificial Intelligence (AI)

As in other areas of medicine, artificial intelligence may play a role in the management of bariatric surgery patients. For example, AI has been used to predict the risk of gastroesophageal reflux disease in patients undergoing SG with good accuracy, though only moderate sensitivity and specificity showed there was room for further development of the model [81]. Other innovations with artificial intelligence have been developed with the same goal: to predict complications and side effects of undergoing bariatric surgery. Similarly, a research group developed a model to predict post-operative nausea and vomiting with notable ability for predicting such sequelae [82]. It is possible that these AI tools may be useful in their ability to predict individuals who may need more careful attention following bariatric surgery to look for complications, as well as assisting in differential diagnoses in the post-operative period. However, further research and innovations may be developed to create a model useful in this regard and across various surgery types.

In endoscopy, artificial intelligence has been recently utilized for more in-depth detection of abnormal findings. For example, computer-aided detection (CADe) systems have been implemented to help detect metaplasia and neoplasia of Barrett’s esophagus, as well as improved detection of abnormalities throughout the upper gastrointestinal tract, which may be a useful tool in diagnostic endoscopy following bariatric surgery [83]. The use of artificial intelligence in the field of post-bariatric endoscopy is limited and warrants further exploration to improve accurate diagnosis and assist in therapeutic endoscopy.

### 6.2. Endoscopic Weight Loss Therapy

Currently, there are two modalities for weight loss therapy that can be performed endoscopically, recognized by the U.S. Food and Drug Administration, which include intragastric balloon and endoscopic sleeve gastroplasty (ESG). Intragastric balloon procedures are characterized by the endoscopic placement of a saline-filled balloon into the stomach, which has been shown to have significant improvement in weight loss in conjunction with lifestyle modification in comparison to a lifestyle approach alone [82]. ESG is characterized by the reduction in gastric volume through the technique of endoscopic gastric suturing; similarly to the intragastric balloon, it has been shown to improve weight loss when combined with lifestyle modification in comparison to weight loss alone [84]. However, when comparing endoscopic weight loss therapy to bariatric surgery, although endoscopic techniques have been shown to have a lower risk of complications, bariatric surgery is superior in terms of long-term weight loss and metabolic remission [85].

## 7. Limitations

This review is subject to several limitations that reflect the current state of the available literature. Much of the evidence supporting endoscopic evaluation and management of post-bariatric surgery complications is derived from retrospective studies, single-center experiences, and case series. As a result, some of the reported outcomes are susceptible to selection bias, institutional practice variation, and limited generalizability. Additionally, differences in study design, definitions of technical and clinical success, follow-up duration, and reported measures make direct comparisons between studies challenging.

The evidence is also heterogeneous across both endoscopic interventions and disease processes. Although endoscopic techniques such as balloon dilation, stenting, endoscopic suturing, over-the-scope clipping, and EVT have demonstrated favorable outcomes in appropriately selected patients, high-quality prospective comparative studies remain limited. Consequently, evidence comparing the indications, patient selection, long-term durability, recurrence rates, adverse events, and cost-effectiveness across these modalities is lacking. Future multicenter prospective studies and randomized comparative trials are needed to better define optimal patient selection, establish standardized treatment algorithms, and clarify the relative roles of available endoscopic therapies in the management of post-bariatric surgery complications.

## 8. Conclusions

As surgical intervention continues to be a mainstay approach to medical management of obesity, it is critical to be cognizant of potential complications of such procedures, to understand the general approach to diagnosis and management of such conditions, and the utility of endoscopic evaluation and intervention, including timing and type. Surgical or radiologic interventions should be prioritized over endoscopic evaluation in patients who are hemodynamically unstable and need emergent treatment. Multidisciplinary teams remain a focus of appropriate management in the post-surgical period, including gastroenterology, nutrition, and other team involvement in conjunction with the surgical team. Future approaches may show increased utilization of artificial intelligence, varying endoscopic interventions and techniques, as well as a rise in endoscopic weight loss interventions as the primary weight management procedure.

## Figures and Tables

**Table 2:** Minimally invasive approaches to address complications of bariatric surgery and their respective uses.

Minimally Invasive Approach to Address Complications of Bariatric Surgery Post-Operatively	Use for Minimally Invasive Approach	Advantages
Stenting and balloon dilation	To address anastomotic strictures, leaks, and fistula control	Higher technical and clinical success rates, low complication rates, and avoidance of surgery
Endoscopic suturing	Revision of anastomoses, and to a lesser extent, closure of fistulae and perforations	Minimally invasive, high technical and clinical success, high closure strength (compared to over-the-scope clipping)
Over-the-scope clipping	Revision of fistulas that do not close with conservative treatment, stenting, balloon dilation, etc.	Minimally invasive, rapid, single-deployment closure, low adverse events, high technical and clinical success
Endoscopic vacuum therapy (EVT)	Closure of intra-abdominal and intrathoracic cavities from perforations, leaks, and fistulae	Multimodal physiologic benefit with a negative pressure mechanism

## Data Availability

No new data were created or analyzed in this study. Data sharing is not applicable to this article.

## Abbreviations

The following abbreviations are used in this manuscript:

RYGB	Roux-en-Y gastric bypass
SG	Sleeve gastrectomy
DS	Biliopancreatic diversion with duodenal switch
BMI	Body mass index
CT	Computed tomography
GI	Gastrointestinal
MU	Marginal ulcer
GJ	Gastrojejunal
GEJ	Gastroesophageal junction
GG	Gastro-gastric
SIBO	Small intestinal bacterial overgrowth
BE	Barrett’s esophagus
ASGE	American Society of Gastrointestinal Endoscopy
TPN	Total parenteral nutrition
EHS	Endoscopic hand suturing
POEM	Peroral endoscopic myotomy
EVT	Endoscopic vacuum therapy
NGT	Nasogastric tube
EUS	Endoscopic ultrasound
ERCP	Endoscopic retrograde cholangiopancreatography
EDGE	EUS-directed trans-gastric ERCP
AGA	American Gastroenterological Association
AI	Artificial intelligence
CADe	Computer-aided detection
ESG	Endoscopic sleeve gastroplasty
